# Editorial: Recent Advances in G Protein-Coupled Receptor Signalling: Impact of Intracellular Location, Environment and Biased Agonism

**DOI:** 10.3389/fphar.2021.707393

**Published:** 2021-05-28

**Authors:** Michelle L. Halls, Anthony P. Davenport, Roger J. Summers

**Affiliations:** ^1^ Drug Discovery Biology Theme, Faculty of Pharmacy and Pharmaceutical Sciences, Monash Institute of Pharmaceutical Sciences, Monash University, Parkville, VIC, Australia; ^2^ Experimental Medicine and Immunotherapeutics, Addenbrooke’s Centre for Clinical Investigation, University of Cambridge, Cambridge, United Kingdom

**Keywords:** GPCR, cell signalling, drug discovery, allosteric action, biased signalling

G protein-coupled receptors (GPCRs), the largest family of cell surface receptors, are targeted by approximately 35% of approved drugs that treat a wide variety of diseases including those of the cardiovascular, respiratory, metabolic, and reproductive systems, and of the central, and peripheral nervous system. Currently the great majority of approved drugs interact with orthosteric binding sites on the receptor as agonists, partial agonists, antagonists or inverse agonists. Of some 800 GPCRs only about 12% have been utilised therapeutically with the remainder being either olfactory or orphan receptors with many having poorly understood signalling mechanisms.

Drug development involving GPCRs in the early stages often utilises recombinant systems that tend to assume that cellular environment has little influence on response. However recent work suggests that membrane phospholipids, sphingolipids, glycolipids and sterols, and in particular cholesterol, influence membrane fluidity and GPCR function. Regions of the plasma membrane that are enriched in combinations of these substituents form microdomains containing not only receptors but other signalling proteins. The plasma membrane organisation of particular cells can therefore have a profound effect on GPCR function and regulation. Another paradigm that is being challenged is that GPCRs are cell surface receptors; many studies now show that GPCRs, their cognant G-proteins, and second messengers, are present, and functional at a number of intracellular sites such as the endoplasmic reticulum where they are synthesised, folded, modified, and assembled but they are also found in nuclear membranes, vesicles, mitochondria, and in the nucleoplasm.

There continues to be great interest in exploiting novel drug binding sites as identified by allostery, oligomerisation, and biased agonism as paradigms for novel drug discovery. Allosteric ligands recognise a topographically distinct site that may be conformationally linked to the orthosteric binding site. Oligomerisation of GPCRs may create new druggable surfaces that discriminate oligomerised receptors from monomeric receptors. Biased agonists may interact with the orthosteric binding site to produce subtly different receptor conformations that differentially affect signalling pathways. Targeting novel drug binding sites has application for studies of orphan GPCRs, for known target receptors where selectivity has not been achieved by conventional approaches, and for receptor targets currently exploited therapeutically but where side effects are a significant problem. These new paradigms are altering approaches to drug development, and have the potential to produce more efficacious drugs with novel modes of action, and reduced unwanted effects.

**FIGURE 1:**
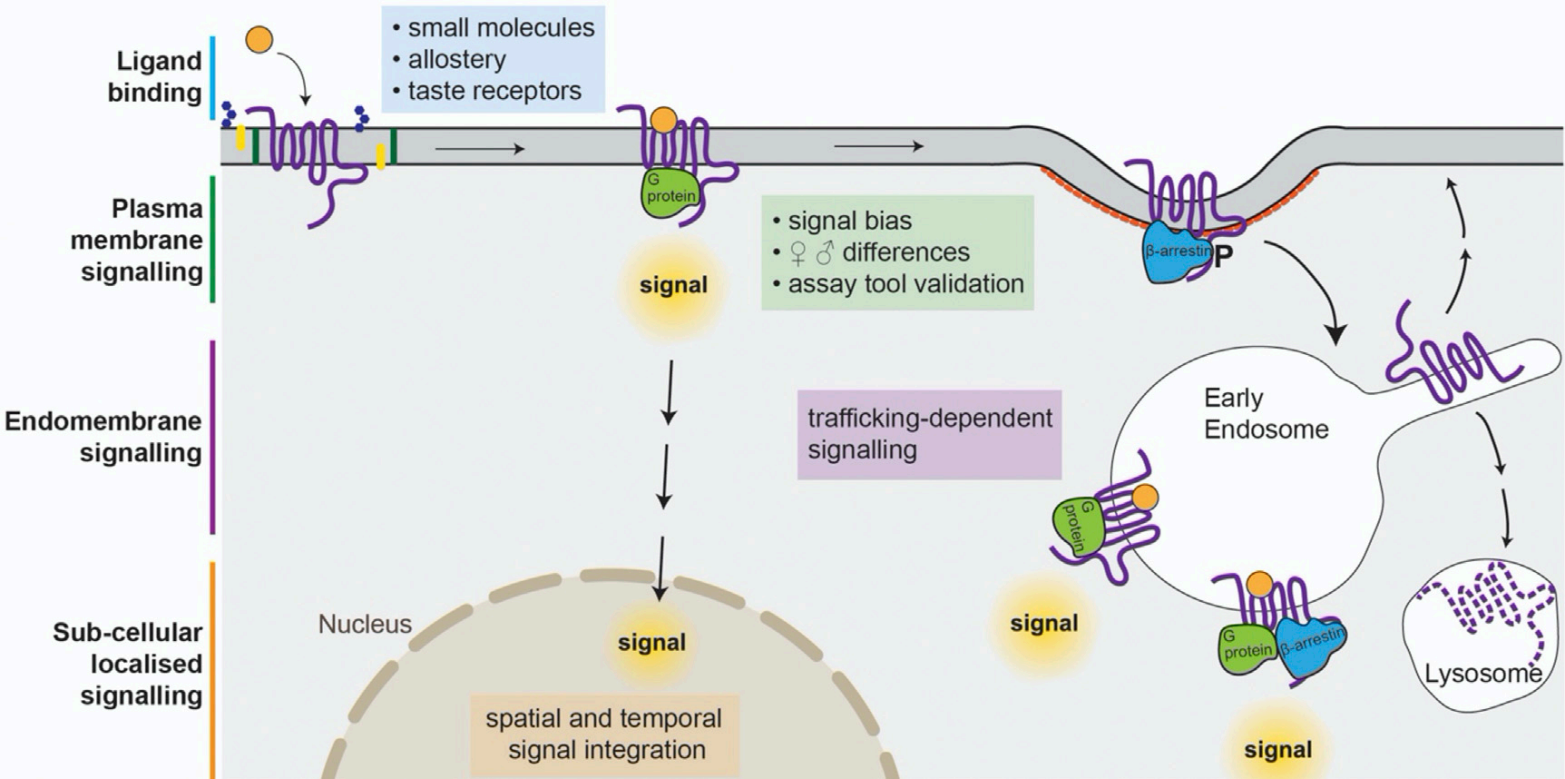
Factors affecting signalling in response to activation of GPCRs. Glycolipids (blue hexagons), phospholipids (green rectangles), and sterols (yellow) can influence ligand binding at orthosteric, and allosteric sites. Ligands can produce different receptor conformations leading to different signalling patterns and degrees of interaction with β-arrestins, internalisation, and desensitisation. Ligands with differing physicochemical properties can result in altered spatial and temporal signal integration, and affect the relative degree of cell surface/intracellular signal transduction as well as influencing receptor recycling or lysosomal degradation.

The studies described in this issue explore many of these concepts. In “Fine tuning muscarinic acetylcholine receptor signalling through allostery and bias”, van der Westhuizen et al. provide an excellent example of known drug targets, the M1 and M4 muscarinic acetylcholine (mACh) receptors, that have been difficult to target selectively using the orthosteric ligand binding site for acetylcholine that is well conserved across mACh receptor subtypes. Their findings suggest that in addition to an allosteric mode of action, drug candidates may have to exhibit a particular bias profile in order to display therapeutic efficacy while minimising on-target side effects. The concept is continued in two studies that examine the utility of allosteric agonists at the Follicle Stimulating Hormone (FSH) receptor. In “Pharmacological programming of endosomal signaling activated by small molecule ligands of the follicle stimulating hormone receptor”, Sposini et al. examine the properties of two low MWt allosteric agonists of the FSH receptor and show that the compounds possessed different abilities to alter receptor endosomal trafficking and cAMP signalling suggesting that these functions can be selected for pharmacologically. In “Discovery and preclinical development of orally active small molecules that exhibit highly selective follicle stimulating hormone receptor agonism”, Nataraja et al. describe the development of orally active FSH receptor allosteric agonists with clinical potential for the treatment of infertility. They show that these compounds mimic the biological activity of recombinant human FSH in preclinical studies, and cause folliculogenesis, and superovulation in rats, and mice. Importantly, the compounds have properties that make them suitable for oral administration, that would avoid the current limitation in the clinic of using recombinant FSH that has to be injected.

The concept of biased agonism is explored in “The G protein biased small molecule apelin agonist CMF-019 is disease modifying in endothelial cell apoptosis *in vitro* and induces vasodilatation without desensitisation *in vivo*” by Read et al. that describes a study on the apelin receptor, a target for the treatment of pulmonary arterial hypertension. The endogenous peptide agonists are unsuitable therapeutics due to poor bioavailability, rapid desensitisation, and rapid inactivation. In contrast, the small molecule agonist CMF-019 not only retains the vasodilator properties of the peptides without receptor desensitisation but protects endothelial cells from apoptosis. Such biased agonism could form the basis for new therapeutic agents. The study on “The effect of cell surface expression and linker sequence on the recruitment of arrestin to the GIP receptor”, by Al-Sabah et al. examines one of the problems associated with the demonstration of bias, that is the choice, and configuration of the assays used. They show that replacement of the native signal peptide in the GIP receptor with a HA signal peptide influences the experimental result probably by increasing receptor cell surface expression. The linker between the receptor and fluorescent protein was also shown to be important and could result in the inclusion of phosphorylation sites that produced false positive results. In another study that points to the care that must be exercised with the use of tool compounds to examine GPCR signalling, Chen and Sabatini caution on “The kinase specificity of protein kinase inhibitor peptide (PKI)” showing in a study of 55 mouse kinases that in addition to inhibition of PKA, PKI also can activate multiple isoforms of PKC. This may suggest re-evaluation of some studies using PKI as a specific inhibitor of PKA.

In “G protein-coupled receptors in taste physiology and pharmacology” Ahmad and Dalziel examine the interesting properties of GPCR taste receptors that not only respond to a wide variety of ligands including orthosteric agonists, and antagonists but also positive, and negative allosteric modulators. While initially identified as taste receptors, it is now recognised that these receptors control other physiological processes in addition to taste, with expression of mRNA, and receptor protein identified in range of tissues in humans (Human Protein Atlas https://www.proteinatlas.org/). They outline the value of potential structural studies but emphasise the challenges associated with these studies and taste receptors and indicate how these may be overcome. The paper by Mouat et al. “Deletion of orphan G protein-coupled receptor GPR37L1 in mice alters cardiovascular homeostasis in a sex-specific manner” provides more evidence that the orphan GPCR GPR37L1 is involved in maintaining sympathetic vasomotor tone. The authors were also able to demonstrate gender differences between responses with female GPR37L1-/- mice showing an attenuation of cardiovascular reactivity to aversive but not appetitive stimuli. The study draws attention to gender differences in physiological responses and how these can affect drug action. Substantial progress has been made in understanding the physiological role of GPR37L1 by using gene deletion and identifying a potential drug target without knowing the identity of the endogenous ligand. The strategy described has wider applicability to the remaining orphan GPCRs that have not yet been exploited.

In their paper “Inhibition of the proliferation of human lung fibroblasts by prostacyclin receptor agonists is linked to a sustained cAMP signal in the nucleus” Roberts et al. (2021), examine factors underlying the anti-fibrotic effects of GPCR agonists acting to increase cAMP. They show that the anti-fibrotic effects of a Gαs-coupled prostacyclin receptor requires sustained increases in cAMP in the nucleus to inhibit PDGF driven nuclear ERK1/2 and fibroblast proliferation indicating that both location and duration of the signal are key factors.

The studies included in this themed issue describe some of the concepts influencing current GPCR research with approaches such as the targeting of allosteric sites and development of biased agonists likely to produce new therapeutics that either act at known targets with fewer side effects or at targets for which there are no drugs currently available. Some studies also point to deficiencies in current assay tools that can be used to improve drug development.
